# Relaxin Protects Astrocytes from Hypoxia *In Vitro*

**DOI:** 10.1371/journal.pone.0090864

**Published:** 2014-03-05

**Authors:** Jordan M. Willcox, Alastair J. S. Summerlee

**Affiliations:** Department of Biomedical science, University of Guelph, Guelph, Ontario, Canada; Hosptial Infantil Universitario Niño Jesús, CIBEROBN, Spain

## Abstract

The peptide relaxin has recently been shown to protect brain tissues from the detrimental effects of ischemia. To date, the mechanisms for this remain unclear. In order to investigate the neuroprotective mechanisms by which relaxin may protect the brain, we investigated the possibility that relaxin protects astrocytes from hypoxia or oxygen/glucose deprivation (OGD). Cultured astrocytes were pre-treated with either relaxin-2 or relaxin-3 and exposed to OGD for 24 or 48 hours. Following OGD exposure, viability assays showed that relaxin-treated cells exhibited a higher viability when compared to astrocytes that experienced OGD-alone. Next, to test whether relaxin reduced the production of reactive oxygen species (ROS) astrocytes were exposed to the same conditions as the previous experiment and a commercially available ROS detection kit was used to detect ROS production. Astrocytes that were treated with relaxin-2 and relaxin-3 showed a marked decrease in ROS production when compared to control astrocytes that were exposed only to OGD. Finally, experiments were performed to determine whether or not the mitochondrial membrane potential was affected by relaxin treatment during 24 hour OGD. Mitochondrial membrane potential was higher in astrocytes that were treated with relaxin-2 and relaxin-3 compared to untreated OGD-alone astrocytes. Taken together, these data present novel findings that show relaxin protects astrocytes from ischemic conditions through the reduction of ROS production and the maintenance of mitochondrial membrane potential.

## Introduction

Cerebral ischemia induces the loss or reduction of oxygen and glucose delivery to brain region affected causing disruption in production of adenosine triphosphate [1], increased reactive oxygen species (ROS) production [2], [3] and sparking inflammatory cascades [4] that may culminate in the death of both neurons and astrocytes. At the core of the infarct zone, near complete death is observed through necrosis within minutes [5], however the region surrounding this core (known as the ischemic penumbra) is partially perfused and does not immediately experience irreversible damage [6]. Within the ischemic penumbra, maintenance of astrocyte viability is critical since neurons are dependent on close interactions with astrocytes for survival [7]. In fact, astrocyte survival can promote synaptic remodeling and neurite outgrowth to compensate for neurons lost through the ischemic insult [7]. Several studies have reported that astrocytes may be particularly susceptible to ischemia. Astrocytes exposed to ischemic challenges experience loss of astrocyte marker proteins and evidence of astrocyte cell death prior to histologic evidence of neuronal death has been observed [8], [9]. Furthermore, prolonged astrocyte survival in areas of cerebral infarction can contribute to protecting neurons from cell death by means of astrocyte-mediated glutamate clearance [10], [11], astrocyte release of metabolic intermediates such as lactate, alanaine, citrate and α-ketogluterate [12]–[16] and finally through scavenging of ROS, particularly through glutathione [17], [18].

Relaxin is a peptide hormone with many diverse actions in multiple tissues [19]. Whilst classically thought of as a hormone of female reproduction, the fact that it is present in the male [20], [21] and has actions outside of the reproductive system [22]–[24], indicates the dogma no longer stands. In addition to the many physiological actions of relaxin that have been reported, relaxin has been shown to protect tissues from ischemia, particularly in models of myocardial infarction [25] and the brain [26]–[28].

Wilson et al. (2006) reported that intracerebral injection of relaxin directly into the cortex prior to middle cerebral artery occlusion (MCAO) reduced ischemic cerebral lesion size indicating a direct action of relaxin on cells of the brain. This group also reported that inhibition of nitric oxide synthase (NOS) blocked this response, implicating nitric oxide (NO) in this observation. These neuroprotective mechanisms may be due to local vasodilation induced by relaxin. However it is also possible that relaxin is acting directly to protect neural tissues and other neuroprotective actions may be possible; experiments from this laboratory on cultured brain slices indicated that in slices exposed to hypoxic conditions, relaxin prevented cell death [28]. Given that these experiments were devoid of a functional circulation, the results show relaxin may have a direct, neuroprotective effect.

In the current study, the direct effect of relaxin on astrocytes in an *in vitro* model of hypoxia was examined. It was hypothesized that relaxin peptides would prevent the production of ROS and thus protect astrocytes from cell death that normally arise from hypoxic conditions. Two types of relaxin, relaxin-2 and relaxin-3 as well as a relaxin chimera peptide, R3/I5, were used in these experiments. Relaxin-2 was used since other reports used this form of relaxin in MCAO or brain slice studies [27], [28]. In addition, relaxin-3, the most recently discovered relaxin-family peptide with nearly exclusive expression in the brain [29] was employed to determine whether or not this peptide provided neuroprotection to astrocytes during hypoxia. Last, since relaxin-3 has been reported to act through both relaxin family peptide receptor (RXFP) 1 and RXFP3, a highly selective RXFP3 agonist, termed R3/I5, was used to elucidate whether or not RXFP3 was involved in relaxin-mediated neuroprotection [30], [31].

## Materials and Methods

### Primary Astrocyte Cell Culture

Primary rat cortical astrocytes were obtained from Invitrogen (Carlsbad, CA, USA) and stored in liquid nitrogen until use. On the day of establishment, vials containing 1×10^6^ cells were thawed and suspended in astrocyte growth media warmed to 37°C; the astrocyte growth media consisted of DMEM 1x (containing 4500 mg/L glucose, 110 mg/L pyruvate, 584 mg/mL L-Glutamine), 15% FBS, and PenStrep (500 units penicillin, 500 µg streptomycin). Cells were plated on 25 cm^2^ tissue culture-treated flasks at a seeding density of 2×10^4^ cells/cm^2^. Flasks were then placed in a water-jacketed incubator at 37°C, 5% CO_2_ and 90% humidity. Fresh, pre-warmed media was replaced every 4 days and cells were grown to 100% confluence.

### Hypoxia Induction Protocols

Astrocytes were cultured in 96-well plates (for viability assays) or 24-well plates (for imaging assays) and grown to 100% confluence. On the day of the experimental protocol, the astrocyte growth media was aspirated and replaced with serum-free, glucose-free media (subsequently referred to as oxygen-glucose deprivation; OGD). Astrocytes were either exposed to untreated, glucose-free media or glucose-free media containing relaxin. The astrocytes were then placed in a polycarbonate hypoxia induction chamber (Stemcell Technologies); a gas mixture containing 5% CO_2_ and 95% N_2_ was used for 10 minutes to purge the ambient air from the chamber and to simulate an ischemic environment. To ensure that the chamber remained humidified throughout the hypoxia protocol, 20 mL of sterile water in a Petri dish was placed in the hypoxia chamber. The hypoxia chambers were sealed, then placed in a 37°C incubator for 12, 24 or 48 hours depending on the experimental protocol. All experiments were repeated nine times (n = 9).

### Assessment of Astrocyte Viability

Astrocytes were incubated with serum-free glucose-free media alone or serum-free glucose-free media containing relaxin. The concentrations of relaxin used in these studies were: relaxin-2 (10, 50 ng/mL), relaxin-3 (10, 50 ng/mL) and a highly selective RXFP3 agonist, R3/I5 (10, 50 ng/mL). Astrocyte cell viability was assessed at 12, 24 and 48 hours by the reduction of 3-(4,5-dimethylthiazole-2-yl)-2,5-diphenyltetrazo-lium bromide (MTT) [32]. Briefly, stock concentrations of MTT (5 mg/mL) were diluted (1∶10) in each well of a 96-well plate. The MTT was incubated with the cells for 30 minutes at 37°C and the reduced formazen product was lysed from the cells using a 100% dimethylsulfoxide solution (DMSO). Absorbance was subsequently measured at 570 nm using a fluorescent microplate reader.

### Reactive Oxygen Species Detection Assay

In order to determine whether or not relaxin peptides prevented the excessive production of ROS as a result of ischemic challenge, ROS were measured using the Image-iT LIVE Green Reactive Oxygen Species Detection Kit. This kit uses a fluorogenic marker, 5-(and-6-)-carboxy-2′,7′-dichlorodihydrofluorescein diacetate (carboxy-H_2_DCFDA) to detect ROS in live cells. Astrocytes were plated on 24-well tissue culture treated plates, containing cover slips, and exposed to either serum-free glucose-free media alone or serum-free glucose-free media with relaxin-2 (10 ng/mL) or relaxin-3 (10 ng/mL) or R3/I5 (10 ng/mL). The astrocytes were then exposed to the hypoxia protocol described above for 12 or 24 hours. At the conclusion of the hypoxia protocol, astrocytes were washed once with warm PBS and then covered with 25 µM carboxy-H2DCFDA working solution; the astrocytes were incubated in this solution for 30 minutes at 37°C in the dark. After 30 minutes, the astrocytes were washed three times in warm PBS and mounted on glass slides. The live astrocytes were imaged immediately using an Olympus IX8I live-cell microscope using filters optimized for fluorescein at 20x. Images were processed using ImagePro Plus Version 5.

### Assessment of Astrocyte Mitochondrial Membrane Potential

The production of ROS under oxidative stress is closely linked with disruptions in mitochondrial membrane potential (Δψ_m_). Therefore, an investigation was undertaken in order to determine whether or not relaxin-treated astrocytes exhibited differences in Δψ_m_ in response to hypoxic challenge. The cationic dye, 5,5′,6,6′-tetrachloro-1,1′,3,3′-tetraethylbenzimidazolcarbocyanine iodide (JC-1) was used to assess Δψ_m_. JC-1 is a cationic dye that exhibits a potential-dependent accumulation in mitochondria detected by a shift in fluorescence from green (∼525 nm) to red (∼590 nm) [33], [34]. Consequently a decrease in the red fluorescence and an increase in green fluorescence indicate the presence of depolarized mitochondrial membranes.

Astrocytes were plated on 24-well tissue culture treated plates, containing cover slips, and exposed to either serum-free glucose-free media alone or serum-free glucose-free media with relaxin-2 (10 ng/mL) or relaxin-3 (10 ng/mL) or R3/I5 (10 ng/mL). The astrocytes were then exposed to the hypoxia protocol described above for 12 hours. At the conclusion of the hypoxia protocol, the media was aspirated, astrocytes were washed once with PBS and incubated with PBS containing the JC-1 dye (2 µg/mL) and incubated for 30 minutes at 37°C. The dye was then removed and the astrocytes were washed three times. The astrocytes were imaged using an Olympus IX81 1ive cell microscope optimized for red and green fluorescence. Images were acquired at both wavelengths at a magnification of 20x. Images were processed using ImagePro Plus Version 5.

### Apoptosis Detection Assay

The detection of astrocytes undergoing apoptosis that was induced by 24 hours of OGD was detected using a commercially available Annexin V conjugate assay that detects phosphatidylserine (PS) on the outer leaflet of the cell membrane. The assay is built upon the principle that in non-apoptotic cells, PS is located on the cytoplasmic surface of membrane; conversely, in apoptotic cells, PS externalizes to the outer leaflet of the membrane thereby allowing Annexin V to conjugate to the exposed PS. Twenty-four hours following the induction of OGD and treatment with relaxin-2, relaxin-3 R3/I5 or control, astrocytes were incubated with PBS-containing Annexin V (5 µg/mL) for 15 minutes. Astrocytes were then washed with PBS and immediately imaged using an Olympus IX8I live-cell microscope for FITC. Images were acquired at a magnification of 20x. Images were processed using ImagePro Plus Version 5.

### Mechanisms of relaxin-mediated neuroprotection

In order to determine the involvement of NO and PI3K in the neuroprotective effects that were observed by relaxin application the following pharmacological blockers were applied for 15 minutes prior to the induction of OGD and throughout the 24 hour OGD (in addition to relaxin-2 or relaxin-3): one of *N*_ω_-Nitro-L-arginine methyl ester hydrochloride (L-NAME, 1 mM) or 1H-[1], [2], [4] Oxadiazolo[4,3-a] quinoxalin-1-one (ODQ, 100 µM) to block NO and one of wortmannin (WORT, 10 µM) or LY294002 (LY, 10 µM). The concentrations of inhibitors were chosen based on dose responses experiments conducted in the lab (data not shown). Once a concentration of an inhibitor was chosen, the inhibitor was incubated with astrocytes for 24 hours and the viability was assessed with an MTT assay to ensure that it was not cytotoxic.

### Materials and Reagents

Rat primary cortical astrocytes, DMEM, FBS and PenStrep, Image-IT LIVE green reactive oxygen species detection kit, Annexin-V and JC-1 cationic dye were purchased from Invitrogen (Carlsbad, CA, USA) and the MTT reagent was obtained from Sigma Aldrich (Oakville, ON, Canada).

Recombinant human relaxin-2, human relaxin-3 and R3/I5 chimera relaxin peptide was purchased from Dr. John D Wade and Dr. Ross AD Bathgate, Howard Florey Institute, Melbourne, Australia.

All inhibitors (L-NAME, ODQ, WORT and LY) were obtained from Sigma Aldrich, Oakville, Ontario, Canada.

### Data Analysis and Statistics

Experiments were performed nine times (n = 9). Data involving the imaging of cells is representative of typical results obtained from experiments performed. Data (where applicable) are presented as mean ± SEM. Statistical significance was accepted if *P*<0.05. Statistical analysis on the raw data was employed using Graph Pad Prism software (San Diego, CA, USA). Statistical significance was assessed using an ANOVA and a post hoc Tukey test for multiple comparisons.

## Results

### Assessment of Astrocyte Viability Following Hypoxic Challenge

**Figure 1:**
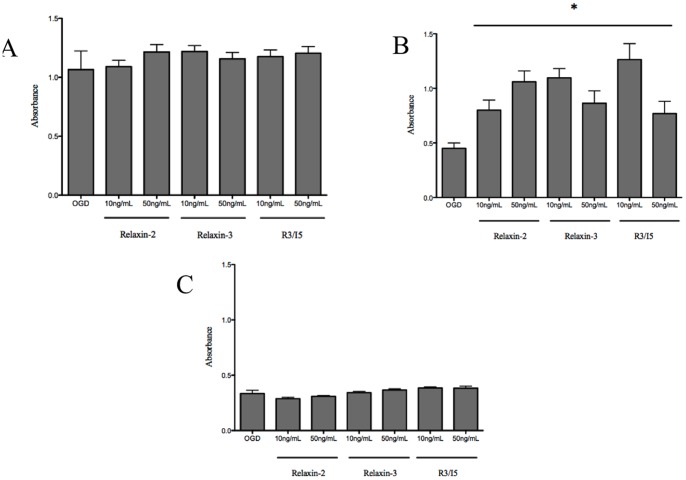
Assessment of astrocyte viability in response to 12 hours of oxygen- glucose deprivation. (**1A**) Astrocyte viability was assessed using an MTT assay and did not show a difference in absorbance between the astrocytes exposed to OGD-alone compared to the astrocytes that were exposed to OGD with relaxin-2 (10, 50 ng/mL), relaxin-3 (10, 50 ng/mL) or R3/I5 (10, 50 ng/mL). (**1B**) Astrocytes that were exposed to relaxin-2, relaxin-3, and R3/I5 during the 24 hour OGD protocol all demonstrated an increased viability when compared to untreated astrocytes that were exposed to OGD for 24 hours. (**1C**) Finally, astrocytes that were exposed to relaxin-2, relaxin-3, and R3/I5 during the 48 hour OGD protocol did not exhibit different viability or mitochondrial uptake of MTT compared to untreated astrocytes that were exposed to OGD. The * indicates significantly different from OGD astrocytes.

The viability of astrocytes in response to OGD challenge was assessed by the uptake of MTT. Exposure of astrocytes to 12 hours of OGD did not cause a significant difference in astrocyte viability between astrocytes that were treated with relaxin-2 (10, 50 ng/mL), relaxin-3 (10, 50 ng/mL) and a highly selective RXFP3 agonist, R3/I5 (10, 50 ng/mL) compared with OGD-alone (Figure 1A).

Astrocytes were exposed subsequently to OGD for 24 hours and treated with media (serum-free, glucose-free)-alone or media with relaxin-2 (10, 50 ng/mL), relaxin-3 (10, 50 ng/mL) and a highly selective RXFP3 agonist, R3/I5 (10, 50 ng/mL). Astrocytes that were treated with relaxin peptides throughout the 24-hour OGD protocol all demonstrated a significant increase in cell viability compared to untreated hypoxic astrocytes (Figure 1B).

Relaxin-2 (10, 50 ng/mL), relaxin-3 (10, 50 ng/mL) and a highly selective RXFP3 agonist, R3/I5 (10, 50 ng/mL) did not protect the viability of astrocytes in response to 48 hours of exposure to OGD *in vitro* (Figure 1C).

### Production of Reactive Oxygen Species by Astrocytes in Response to Oxygen Glucose Deprivation

**Figure 2:**
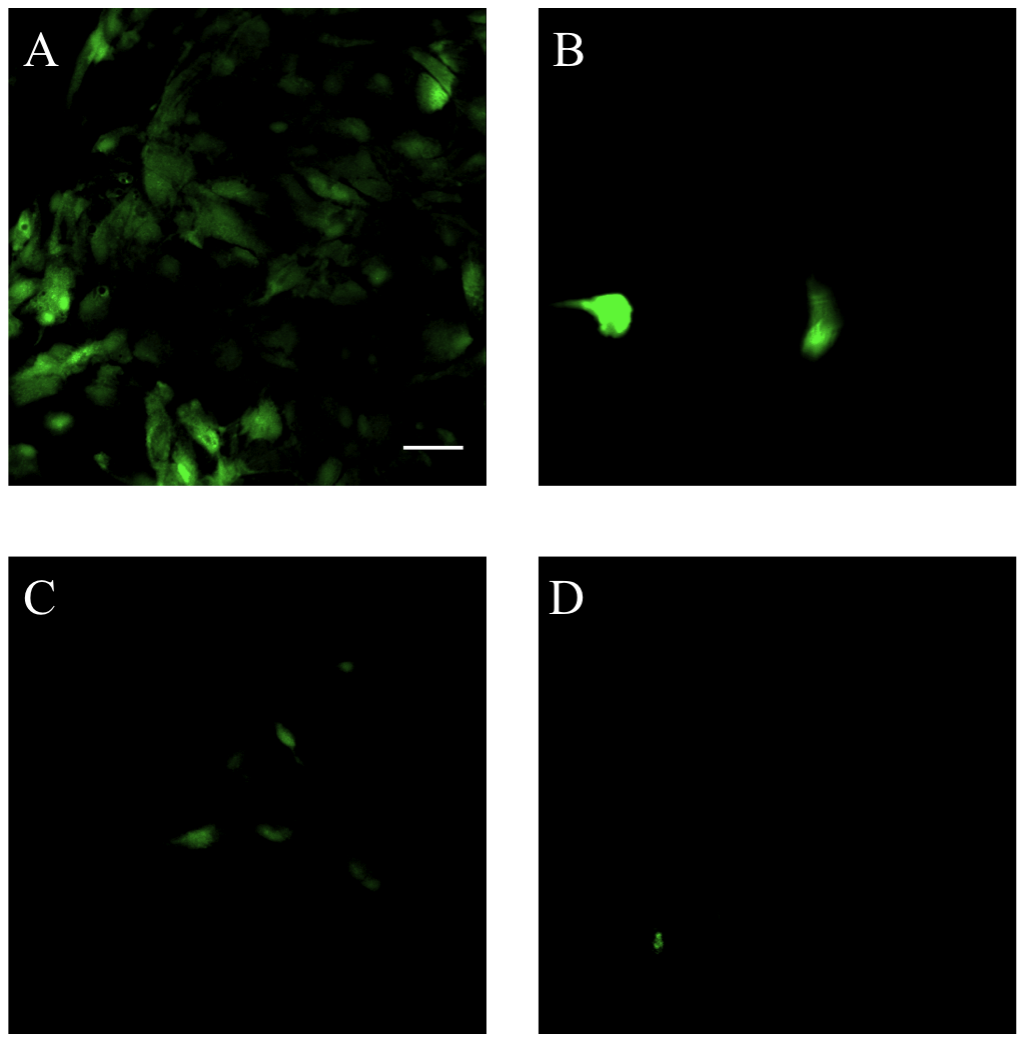
Detection of reactive oxygen species in astrocytes in response to 12 hours of oxygen-glucose deprivation. (A) Astrocytes that were exposed to OGD for 12 hours and treated with only serum-free, glucose-free media demonstrate a strong fluorescent signal indicating the production of ROS in astrocytes in response to OGD. Astrocytes that were treated with 10 ng/mL relaxin-2 (B), 10 ng/mL relaxin-3 (C) or 10 ng/mL R3/I5 (D) show a reduction in the production of ROS in response to OGD compared to untreated astrocytes (A). Scale bar = 50 µm.

The production of ROS by astrocytes in response to OGD was assessed with the fluorogenic marker carboxy-H_2_DCFDA. Astrocytes that were exposed to 12 hours of OGD demonstrated a greater amount of fluorescent signal compared to astrocytes that were treated with relaxin-2 (10 ng/mL), relaxin-3 (10 ng/mL) and a highly selective RXFP3 agonist, R3/I5 (10 ng/mL) indicating that relaxin may prevent the production of ROS in response to OGD over a 12 hour period (Figure 2). As a control, astrocytes that were not exposed to OGD were also loaded with H_2_DCFDA and did not show any signal (data not shown).

**Figure 3:**
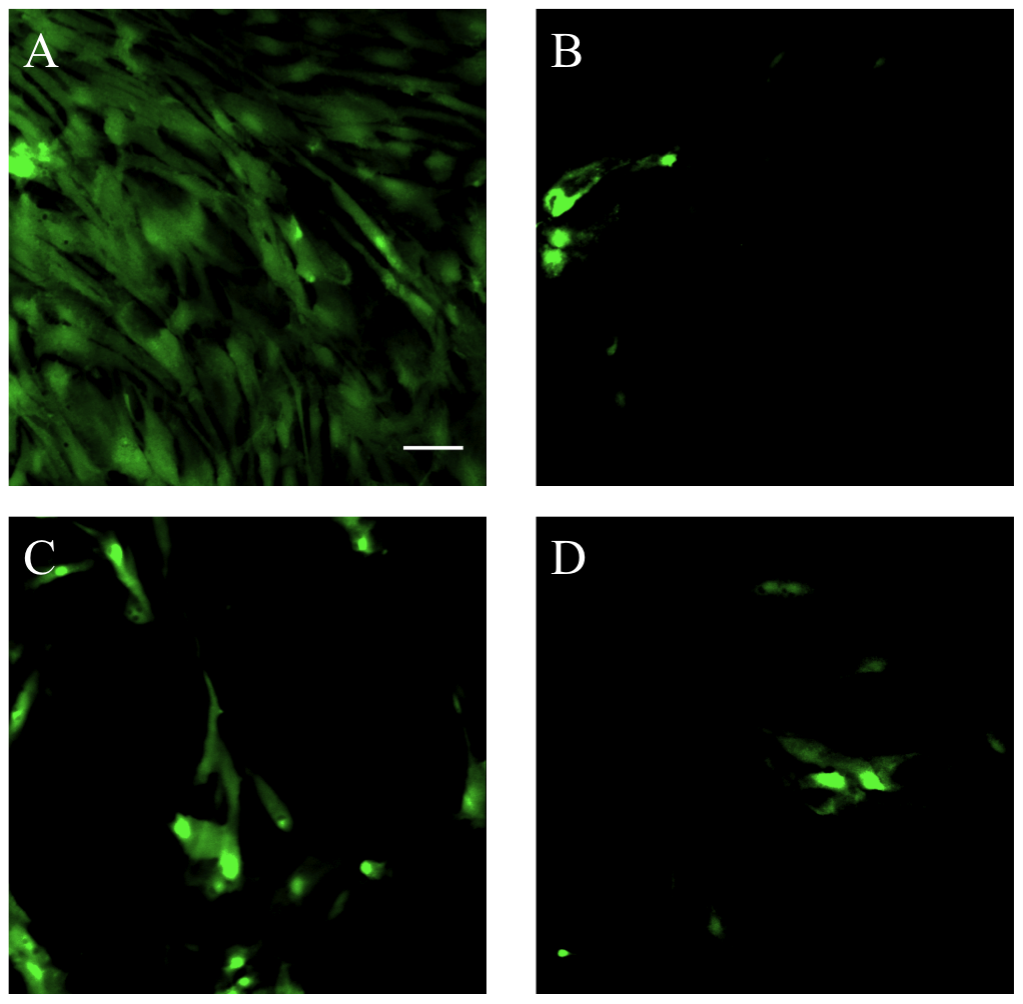
Detection of reactive oxygen species in astrocytes in response to 24 hours of oxygen-glucose deprivation. (A) Astrocytes that were exposed to OGD for 24 hours and treated with only serum-free, glucose-free media demonstrate a strong fluorescent signal indicating the production of ROS in astrocytes in response to OGD. Astrocytes that were treated with 10 ng/mL relaxin-2 (B), 10 ng/mL relaxin-3 (C) or 10 ng/mL R3/I5 (D) show a marked reduction in the production of ROS in response to OGD compared to untreated astrocytes (A). Scale bar = 50 µm.

The production of ROS in astrocytes in response to OGD was also assessed over a 24 hour exposure to OGD. Astrocytes that were treated with media alone exhibited a marked increase in ROS production (indicated by the flourometric carboxy-H_2_DCFDA signal) when compared to those astrocytes that were treated with relaxin-2 (10 ng/mL), relaxin-3 (10 ng/mL) and a highly selective RXFP3 agonist, R3/I5 (10 ng/mL) indicating that relaxin may prevent the production of ROS in response to OGD over 24 hours in astrocytes (Figure 3).

### Assessment of Mitochondrial Membrane Potential in Astrocytes Exposed to 12 Hours of Oxygen-glucose Deprivation

**Figure 4:**
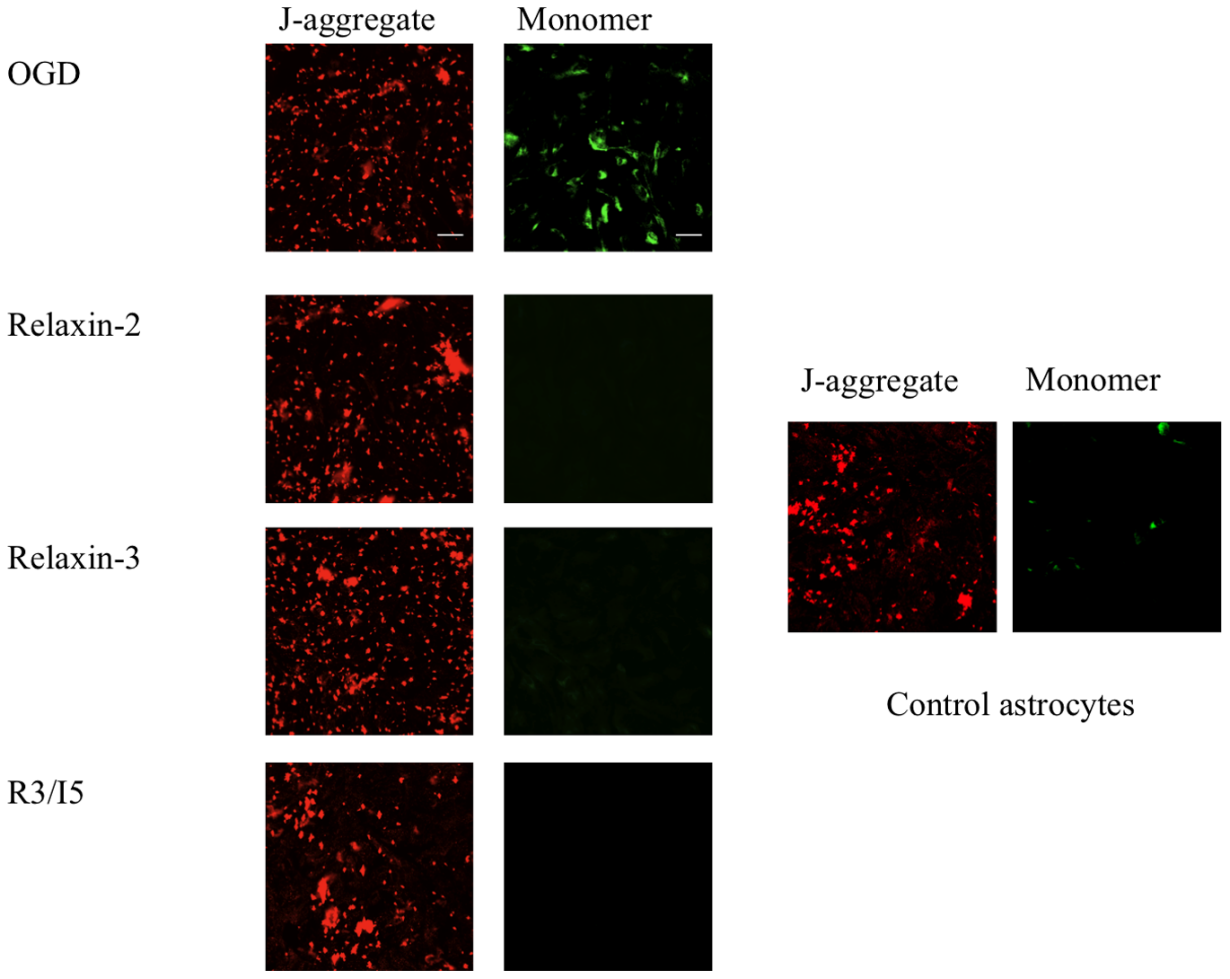
Mitochondrial membrane potential in response to 12 hours of oxygen- glucose deprivation. Astrocytes were exposed to OGD for 12 hours and treated with serum-free, glucose-free media or relaxin-2 (10 and 50 ng/mL), relaxin-3 (10 and 50 ng/mL), and R3/I5 (10 and 50 ng/mL); Δψ_m_ as assessed with JC-1 (2 µg/mL). Decreases in Δψ_m_ are indicated by green fluorescence (monomer) compared with the J-aggregate (red fluorescence). Untreated astrocytes demonstrated an increase in monomer staining compared with relaxin-2, relaxin-3 and R3/I5. Control astrocytes indicate astrocytes that were not exposed to OGD and loaded with JC-1 as a control. Scale bar = 50 µm.

Differences in Δψ_m_ were assessed using the cationic dye JC-1 (2 µg/mL). Staining with JC-1 allows cells to be excited at two different wavelengths in order to assess Δψ_m_ in cells undergoing oxidative stress or other challenges that would affect Δψ_m_ such as apoptosis. Red fluorescence (J-aggregate form, ∼585 nm excitation) indicates polarized mitochondrial membranes whereas green fluorescence (monomer form, ∼514 nm excitation) indicates depolarized mitochondrial membranes. Astrocytes that were treated with relaxin-2 (10 ng/mL), relaxin-3 (10 ng/mL) and a highly selective RXFP3 agonist, R3/I5 (10 ng/mL) exhibited a marked difference in JC-1 staining compared with astrocytes that were solely exposed to OGD (Figure 4). Those astrocytes that were treated with relaxin peptides show marked staining for the J-aggregate form of JC-1 and a limited amount of staining for the monomer form. This compared to astrocytes that were exposed to OGD alone that exhibited much more staining for the monomer form thereby indicating that the mitochondrial membranes of astrocytes that were exposed to OGD are more depolarized compared to that of astrocytes that were treated with relaxin-2 (10 ng/mL), relaxin-3 (10 ng/mL) and R3/I5 (10 ng/mL).

### Apoptosis Detection Assay

**Figure 5:**
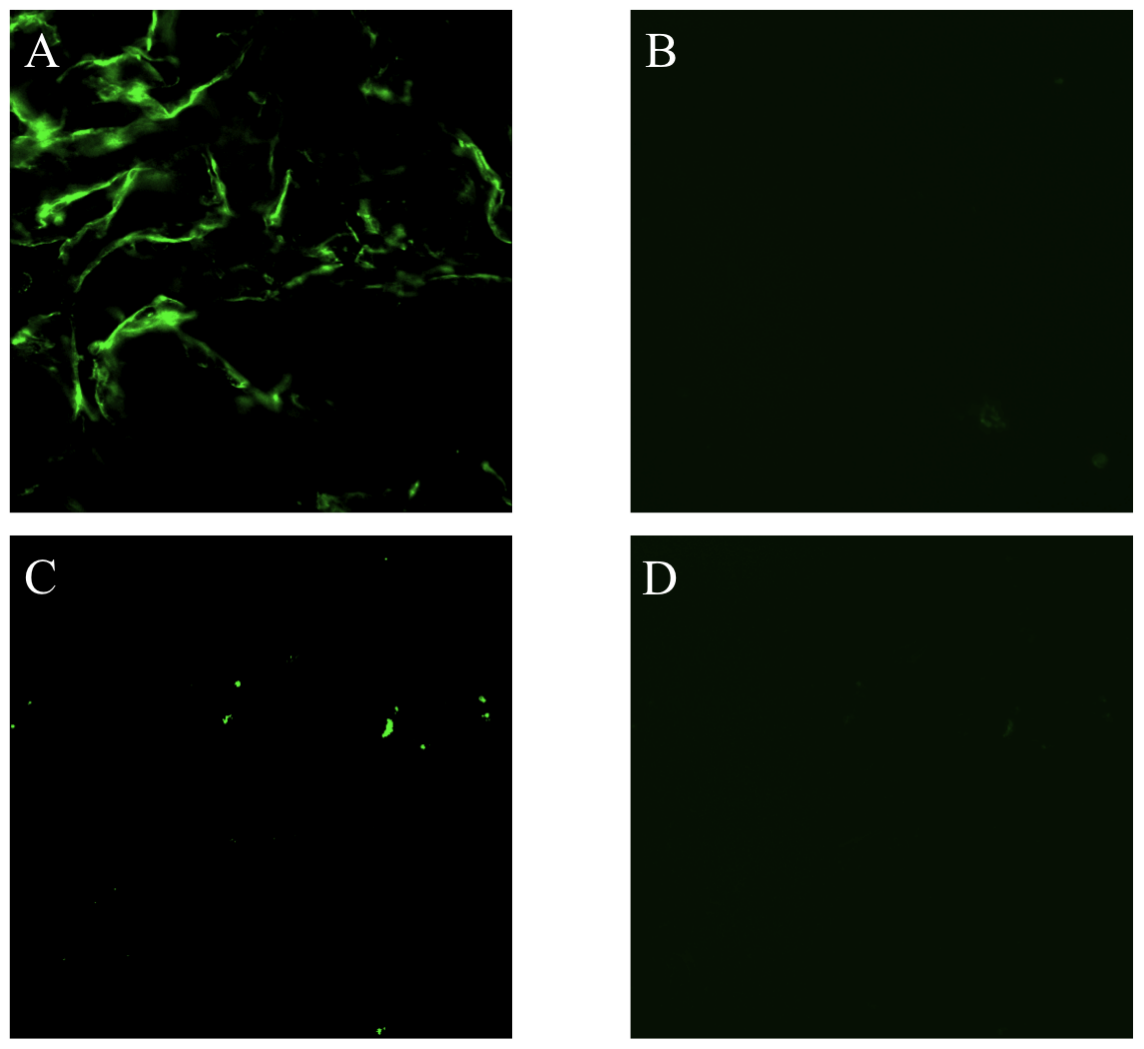
Detection of apoptosis in astrocytes that were exposed to oxygen-glucose deprivation for 24 hours. Apoptosis was induced in cultured astrocytes for 24 hours in an OGD chamber detected using an Annexin-V assay. Astrocytes that were exposed OGD-alone displayed a higher incidence of apoptosis as indicated by green Annexin-V staining along the membranes of the astrocytes. Astrocytes that were exposed to one of relaxin-2, relaxin-3 or R3/I5 and OGD showed a lower incidence of apoptosis.

The identification of astrocytes that were undergoing apoptosis as a result of 24 hour-OGD exposure was examined by loading astrocytes with Annexin V (an early marker of apoptosis through labeling translocated PS) and PI (a marker of cell death). Astrocytes that were not treated with relaxin (Figure 5A) exhibited characteristic Annexin V labeling as indicated by green fluorescence showing an increase in apoptosis in these cells. Astrocytes that were incubated with either relaxin-2, relaxin-3 or R3/I5 (Figure 5B–D) showed a marked decrease in apoptosis that was indicated by a much lower green fluorescence signal.

### Mechanisms of relaxin-mediated neuroprotection

**Figure 6:**
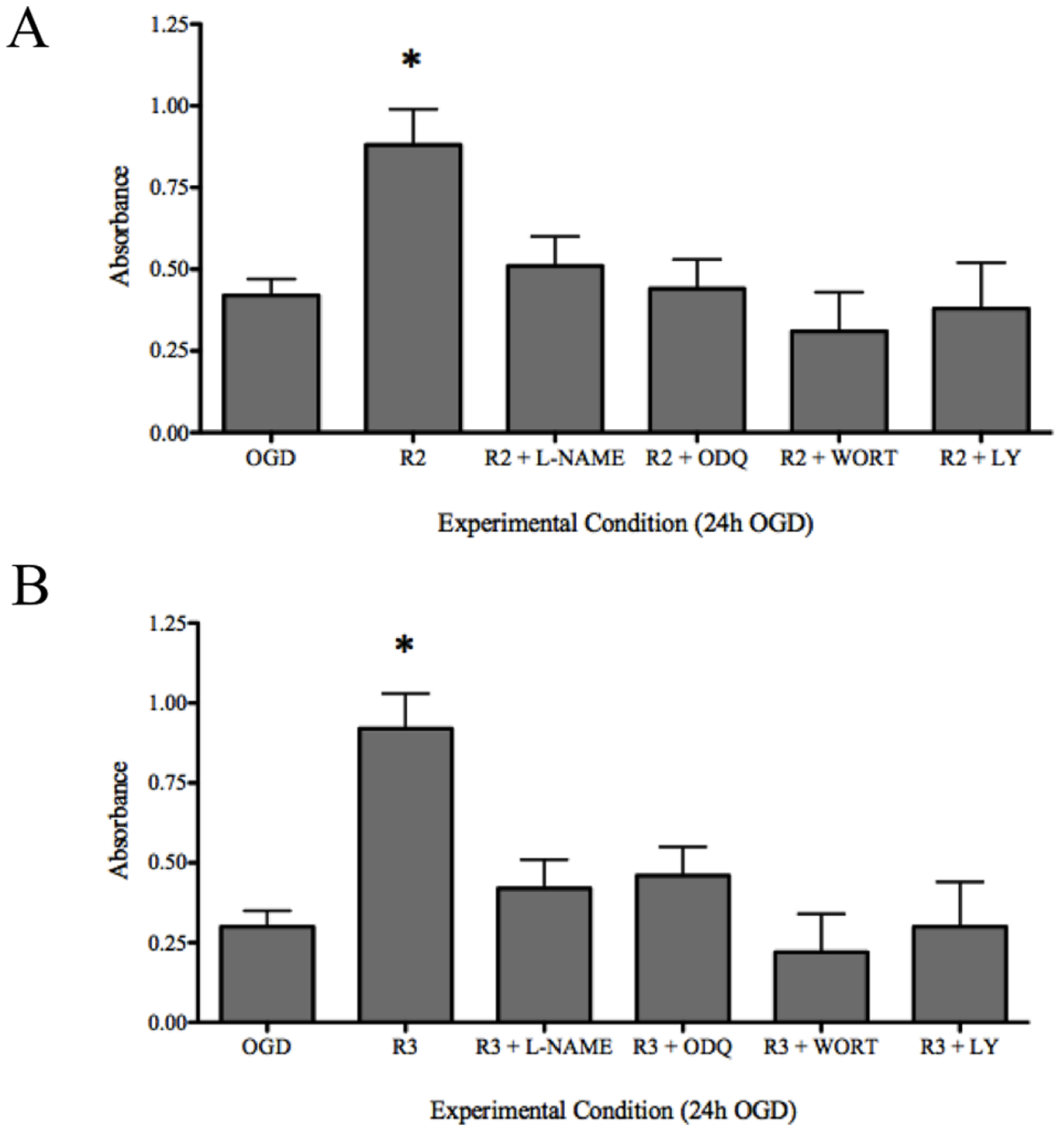
Assessment of astrocyte viability in response to 24 hours of oxygen- glucose deprivation with relaxin and inhibitors of inhibitors of NO and PI3K. (**1A**) Astrocyte viability was assessed with an MTT assay and showed that astrocytes incubated with relaxin-2 had a viability that was significantly higher than astrocytes that were exposed to OGD-alone. This observation was ablated by inhibiters for NO and PI3K. (**1B**) Astrocyte viability was assessed with an MTT assay and showed that astrocytes incubated with relaxin-3 had a viability that was significantly higher than astrocytes that were exposed to OGD-alone. This observation was reduced in cultures that also had inhibiters for NO and PI3K.

In order to determine whether or not NO and PI3K was involved in the protection of astrocyte viability from the effects of 24 hour-OGD, astrocytes were relaxin-2 or relaxin-3 and either L-NAME, OGD (to block NO) or WORT or LY (to block PI3K) during the 24 hour OGD protocol. The results show that the inhibition of NO or PI3K significantly reduced the relaxin-2 (Figure 6A) and relaxin-3 (Figure 6B) protective effects during the 24 hour-OGD protocol.

## Discussion

The objectives of this study were to determine whether or not relaxin-2 and relaxin-3 protected cultured astrocytes from cell death induced by hypoxia *in vitro*, and to investivate whether or not relaxin had an effect on some of the mediators of cellular damage that results from hypoxia, namely the production of ROS and disruption of mitochondrial function. The data presented indicate that relaxin-2, relaxin-3 and R3/I5 protect cultured astrocytes from cell death over a 24 hour period. These data also show that cultured astrocytes that were treated with relaxin-2, relaxin-3 and R3/I5 show a marked decrease in ROS production over the course of 12 and 24 hour exposures to hypoxia. Further, hypoxic conditions that result in mitochondrial depolarization and apoptosis appear to be reduced when astrocytes are incubated with relaxin-2, relaxin-3 and R3/I5 during hypoxic challenge. Finally, we show that the inhibition of NO and PI3K prevents the relaxin-2 and relaxin-3-mediated protection of astrocytes during OGD.

In a number of previous studies, relaxin has been demonstrated to confer protective effects to tissues that are undergoing ischemic stress. Work from Bani and colleagues [25], [35], [36] have provided ample evidence that relaxin protects the heart from ischemia in a number of studies investigating myocardial infarction in guinea pigs and rats [25], [36]. Also in these studies, these researchers have reported on relaxin’s protective effect as a consequence of activation of NOS and the downstream production of NO [36]. Our study also shows that NOS is involved in the protection of astrocytes from the effects of OGD. Relaxin has also been reported to reduce the lesion development as a result of cerebral ischemia [26], [27]. These results show that pre-treatment with relaxin-2 administered intracerebrally reduced the lesion size compared to untreated animals following MCAO. Further work from this laboratory has indicated that brain slices exposed to hypoxia *in vitro* exhibit higher viability when co-incubated with relaxin-2 compared to untreated cells.

The current study provides some possible insight into some of the mechanisms by which relaxin may protect neural tissues from cerebral ischemia. The findings show that relaxin-2, relaxin-3 and R3/I5 protect astrocytes from cell death in an *in vitro* model of hypoxia. Neurons require a close interaction with astrocytes for survival as astrocytes provide a multitude of functions that ensure proper neuronal function (e.g. structural scaffolding, extracellular ion regulation, control of pH, neurotransmitter clearance etc.) and the death of astrocytes would directly impact the fate of neurons. Therefore the protection of astrocytes from cell death may not only directly protect astrocytes but positively influence (protect) the cell death in neurons as well.

In addition to the demonstration that relaxin-2 affords protection to astrocytes that were exposed to a hypoxic challenge, data presented are the first to report that relaxin-3 also has this action. Given that relaxin-3 interacts with both relaxin receptors (RXFP1 and RXFP3) it was important to confirm an involvement of RXFP3 by using a highly selective RXFP3 agonist, R3/I5. In doing so these data show that activation of both RXFP1 and RXFP3 provide protection from hypoxia.

Astrocytes, like other cells and tissues experiencing hypoxia, are vulnerable to the production of ROS that result in cellular damage, impaired cellular function and potentially cell death [37]–[39]. Reactive oxygen species is a phrase used to describe molecules and free radicals (chemical species with one unpaired electron) that are derived from oxygen. Superoxide anion is one of the most common free radical precursors of most ROS produced in cells [40] and is a byproduct of a number of enzymatic complexes of the electron transport chain of the mitochondria [2], [41]–[43]. In the brain, it has been reported that complex I is the primary source of O_2_^−^• [44]. Under basal conditions free radicals are usually scavenged or converted to non-reactive species usually resulting in the formation of water; SODs [45] are the most common means of scavenging O_2_^−^•. Cells that are experiencing oxidative stress produce an imbalance of ROS that overwhelms the endogenous ROS scavenging systems and further oxidative damage resulting in the disruption of cellular proteins [46], lipids [47], polysaccharides [48] and DNA [49].

These data presented in the current paper show that astrocytes treated with relaxin-2, relaxin-3 and R3/I5 show a marked reduction in ROS production over a 12 and 24 hour period. Furthermore, over a 12 hour period, relaxin-2 and relaxin-3 prevented the collapse in the Δψ_m_ when compared to those astrocytes that were exposed to OGD alone. We show that relaxin may be working to protect astrocytes from hypoxic challenges by preventing the production of ROS and affecting (positively) the mitochondrial integrity. We have also shown that two signalling pathways (NO and PI3K) are implicated in the protection of astrocytes during exposure to OGD.

A possible mechanism by which relaxin may be protecting the cell from hypoxia include inducing the expression of hypoxia inducible factor-1 alpha (HIF-1α) through a nuclear factor kappa B (NF-κB) mechanism. Relaxin has been reported to increase the expression of NF-κB [50], [51], which could lead to the production of HIF-1α which helps cells resist damage as a result of hypoxia [52]. Furthermore, induction of NF-κB by relaxin may also affect the regulation and expression of mitochondrial SOD; for example, increased levels of SOD within the mitochondrial matrix could eliminate O_2_^−^• that is formed during hypoxia [45], [53]–[55]. Finally, it has been reported that an increase in the availability of adenosine diphosphate (ADP) within the cytosol directly affects ROS production resulting in a decrease in the Δψ_m_ [2], [56], [57]. Through the numerous phosphorylation events that arise from relaxin-RXFP signaling, ADP levels within the cytosol may increase [58]–[60] and relaxin-induced availability of ADP may directly affect the mitochondrial function of the cells and therefore act in part to prevent apoptosis. Further study is also warranted into the possibility that relaxin peptides mediate protection during reperfusion injury. Other investigations into the ability for relaxin to protect tissues during reperfusion injury have looked at the heart and observed a protective effect [25]. Therefore, a more clear picture would emerge as to the neuroprotective effect of relaxin if ischemia/reperfusion was investigated. Further study on an ischemia/reperfusion *in vivo* model is warranted to investigate this possibility.

Taken together these results presented here provide evidence that relaxin-2, relaxin-3 and an RXFP3-specific agonist, R3/I5 protect astrocytes from cell death induced by OGD. These data also indicate that astrocytes that have been exposed to relaxin peptides exhibited a reduction in the hypoxia-induced production of ROS over a 12 and 24 hour period and more viable mitochondria as shown by a maintenance of Δψ_m_. These data provide insight into the mechanisms by which relaxin may act on astrocytes to provide neuroprotection and presents a possible therapeutic potential to treat this cerebral ischemia and stroke.
